# A First-in-human, Dose-escalation Study of the Methionine Aminopeptidase 2 Inhibitor M8891 in Patients with Advanced Solid Tumors

**DOI:** 10.1158/2767-9764.CRC-23-0048

**Published:** 2023-08-24

**Authors:** Michael A. Carducci, Ding Wang, Christina Habermehl, Matthias Bödding, Felix Rohdich, Floriane Lignet, Klaus Duecker, Oleksandr Karpenko, Linda Pudelko, Claude Gimmi, Patricia LoRusso

**Affiliations:** 1 Oncology and Urology, Hopkins Kimmel Cancer Center, Baltimore, Maryland.; 2 Phase 1 Clinical Trials Program, Henry Ford Cancer Institute, Detroit, Michigan.; 3 Biostatistics, the healthcare business of Merck KGaA, Darmstadt, Germany.; 4 Clinical Pharmacology, the healthcare business of Merck KGaA, Darmstadt, Germany.; 5 Pharmacokinetics, the healthcare business of Merck KGaA, Darmstadt, Germany.; 6 Clinical Biomarkers, the healthcare business of Merck KGaA, Darmstadt, Germany.; 7 Safety Strategy, Olexacon Ltd., London, United Kingdom.; 8 Clinical Development, the healthcare business of Merck KGaA, Darmstadt, Germany.; 9 Medical Oncology, Yale University, New Haven, Connecticut.

## Abstract

Methionine aminopeptidase 2 (MetAP2) is essential to endothelial cell growth and proliferation during tumor angiogenesis. M8891 is a novel orally bioavailable, potent, selective, reversible MetAP2 inhibitor with antiangiogenic and antitumor activity in preclinical studies. The safety, tolerability, pharmacokinetics, and pharmacodynamics of M8891 monotherapy were assessed in a phase I, first-in-human, multicenter, open-label, single-arm, dose-escalation study (NCT03138538). Patients with advanced solid tumors received 7–80 mg M8891 once daily in 21-day cycles. The primary endpoint was dose-limiting toxicity (DLT) during cycle 1, with the aim to determine the maximum tolerated dose (MTD). Twenty-seven patients were enrolled across six dose levels. Two DLTs (platelet count decrease) were reported, one each at 60 and 80 mg/once daily M8891, resolving after treatment discontinuation. MTD was not determined. The most common treatment-emergent adverse event was platelet count decrease. M8891 plasma concentration showed dose-linear increase up to 35 mg and low-to-moderate variability; dose-dependent tumor accumulation of methionylated elongation factor 1α, a MetAP2 substrate, was observed, demonstrating MetAP2 inhibition. Pharmacokinetic/pharmacodynamic response data showed that preclinically defined target levels required for *in vivo* efficacy were achieved at safe, tolerated doses. Seven patients (25.9%) had stable disease for 42–123 days. We conclude that M8891 demonstrates a manageable safety profile, with dose-proportional exposure and low-to-moderate interpatient variability at target pharmacokinetic/pharmacodynamic levels at ≤35 mg M8891 once daily. On the basis of the data, 35 mg M8891 once daily is the recommended phase II dose for M8891 monotherapy. This study forms the basis for future development of M8891 in monotherapy and combination studies.

**Significance:**

M8891 represents a novel class of reversible MetAP2 inhibitors and has demonstrated preclinical antitumor activity. This dose-escalation study assessed M8891 treatment for patients with advanced solid tumors. M8891 demonstrated favorable pharmacokinetics, tumoral target engagement, and a manageable safety profile, and thus represents a novel antitumor strategy warranting further clinical studies.

## Introduction

Methionine aminopeptidase 2 (MetAP2) is one of two cytoplasmic methionine aminopeptidases ubiquitously expressed in mammalian cells. MetAPs catalyze N-terminal methionine excision from newly formed proteins, an essential step in cotranslational protein maturation ensuring protein stability and functionality. MetAP2 has been shown to play an essential role in the growth and proliferation of endothelial cells during tumor angiogenesis and tumor cell proliferation (1, 2).

Thus, MetAP2 could be a promising target in oncology (3–5). The anticancer effect of MetAP2 inhibitors is thought to be a combination of MetAP2 inhibition in endothelial cells (i.e., antiangiogenic) and tumor cells (i.e., antiproliferative; ref. 6). The highly potent fumagillin family of natural, irreversible MetAP2 inhibitors has been shown to block cell-cycle progression and angiogenesis through p53-dependent induction of p21^WAF1/CIP1^ (3, 7–9). However, clinical development of fumagillin derivatives, such as TNP-470, was discontinued because of unfavorable pharmacokinetic profiles and adverse events (10). The toxicity associated with the fumagillin class of MetAP2 inhibitors is theorized to be due to their irreversibility and high systemic exposure necessary to achieve sufficient target coverage (11), rather than inhibition of MetAP2 itself (12). For this reason, several structurally divergent and reversible MetAP2 inhibitors have been developed, including the substrate-like bestatin class (12), the modified marine natural product bengamide class (13), and triazole or purine derivates (1, 14). While several reversible MetAP2 inhibitors are in clinical development, none have so far reached market approval.

M8891 represents a novel class of orally bioavailable, potent, selective, and reversible MetAP2 inhibitors (15, 16). In preclinical studies, M8891 has demonstrated inhibition of new blood vessel formation and tumor cell proliferation and has also shown strong and durable antitumor activity in combination with vascular endothelial growth factor receptor (VEGFR)-targeted tyrosine kinase inhibitors (TKI), such as sunitinib, cabozantinib, and axitinib, in patient-derived renal cell carcinoma xenografts (ref. 17, Friese-Hamim M, personal communication). A substrate of MetAP2, translation elongation factor 1-alpha-1, was recently identified as a pharmacodynamic biomarker to follow target engagement (Friese-Hamim M, personal communication).

We conducted this phase I study to assess the safety, tolerability, pharmacokinetics, and pharmacodynamics of M8891 monotherapy in patients with advanced solid tumors.

## Materials and Methods

### Study Design

This was a first-in-human (FIH), phase I, open-label, multicenter, single-arm, dose-escalation study designed to determine the maximum tolerated dose (MTD), recommended phase II dose (RP2D), safety, tolerability, pharmacokinetic, and pharmacodynamic profiles of M8891 in patients with advanced solid tumors (NCT03138538). An adaptive study design was applied, using a Bayesian two-parameter logistic regression model (BLRM). This study was designed to have two phases. Part 1, the monotherapy, dose-escalation phase, was completed according to protocol and is reported here. Part 2 of the study (combination of M8891 with cabozantinib) was not initiated; this was not due to safety concerns. Twenty-seven patients were enrolled from five sites in the United States between August 2017 (first patient visit) and September 2020 (last patient visit).

This study was conducted in accordance with the protocol and consensus ethical principles derived from international guidelines including the Declaration of Helsinki, applicable International Conference on Harmonization Good Clinical Practice Guidelines, and other applicable laws and regulations. The protocol and all required associated documents were approved by the responsible Institutional Review Board or Independent Ethics Committee. All patients were required to provide written informed consent prior to enrollment.

### Patient Eligibility

Eligible patients were ≥18 years of age and provided written informed consent; had histologically confirmed advanced solid tumors, refractory to or intolerant of existing cancer therapies, with no surgical, radiation, or systemic anticancer therapies available after at least one prior systemic anticancer therapy; had accessible tumor biopsies; and agreed to use a highly effective form of contraception.

Key exclusion criteria included: an Eastern Cooperative Oncology Group (ECOG) performance status ≥2; severe bone marrow (hemoglobin <9.0 g/dL, neutrophil count <1.5 × 10^9^/L, platelets <100 × 10^9^/L), renal (calculated creatinine clearance <60 mL/minute according to the Cockcroft-Gault formula), or liver [total bilirubin >1.5 × upper limit of normal (ULN) or aspartate aminotransferase (AST)/alanine aminotransferase (ALT) >2.5 × ULN (>5 × ULN for patients with liver involvement)] impairment; prior radiotherapy to >30% of bone marrow reserves or bone marrow/stem cell transplantation within 5 years of study start; clinically significant cardiac conduction abnormalities; history of stroke, heart attack, thrombosis [e.g., deep vein thrombosis (DVT) and pulmonary embolism] or genetically determined hypercoagulopathy within 6 months; DVT based on lower extremity screening using Doppler ultrasonography or thromboembolic events under therapeutic anticoagulation; pregnancy or nursing; history of difficulty swallowing, malabsorption, or other chronic gastrointestinal disease or conditions that may have hampered compliance and/or absorption of the investigational drugs; life expectancy <3 months; and known hypersensitivity to the trial treatment or to one or more of the excipients used.

### Treatment

Patients received oral M8891 in 21-day cycles at doses of 7–80 mg once daily. The starting dose of 7 mg once daily was based on the dog highest non-severely toxic dose of 0.75 mg/kg/day divided by a safety factor of 6, which equated to a human dose of 7.5 mg (rounded to 7 mg) for a participant of 60 kg bodyweight. Safety Monitoring Committee (SMC) decisions on subsequent dose levels were supported by a BLRM with overdose control. In addition, the SMC could decide to switch from a once daily to twice daily dosing schedule if data suggested insufficient exposure (pharmacokinetics) and target engagement (pharmacodynamics) after completion of the dose-limiting toxicity (DLT) period of the third once daily dosing cohort (or at a later time) or if evidence suggested that *C*_max_ was driving safety signals, including but not limited to DLTs. Patients received treatment until disease progression, unacceptable toxicity, or withdrawal from the study.

### Assessments and Endpoints

The primary endpoint was DLTs during the first 21-day treatment cycle of M8891, based on a predefined set of treatment-emergent adverse events (TEAE) with the aim to determine the MTD of M8891. At each dose level, the first patient was observed for DLTs for ≥7 days before the dosing of 2 subsequent patients was commenced. DLTs were defined as any of the following adverse events observed during the first 21-day treatment cycle and judged to be M8891-related or clinically relevant: death; events of clinical significance that would expose patients to unacceptable risk if dose escalation continued; treatment-related hepatocellular injury, for example, ALT/AST >3 × ULN with elevation of serum total bilirubin to >2 × ULN, without findings of clinical causality; grade 4 liver enzyme elevation; Grade 4 neutropenia or thrombocytopenia lasting >5 days; grade 3 neutropenia with fever; grade ≥3 thrombocytopenia with bleeding; treatment interruption of >7 days or >30% of total dose due to adverse events not related to the underlying disease or concomitant medication; grade ≥3 non-hematologic toxicity (excluding grade 3 nausea or vomiting lasting <48 hours and resolving to grade ≤1 spontaneously or with conventional medical intervention; grade 3 fatigue or rash of <5 days duration; grade 3 hypertension in the absence of maximal medical therapy; and grade 3 electrolyte abnormality that lasted <72 hours, was not clinically complicated, and resolved spontaneously or responded to conventional medical intervention). In addition, the SMC could define as a DLT any TEAE that impaired daily function, or any abnormality that occurred in patients treated with M8891 at any time in cycle 1 during the dose-escalation part of the trial.

Secondary objectives included evaluating the safety, tolerability, pharmacokinetics, antitumor activity, and determining the RP2D of M8891. Safety evaluations included incidence and severity of TEAEs. All TEAEs were coded according to the Medical Dictionary for Regulatory Activities v23.0 (18) and graded according to the NCI Common Terminology Criteria for Adverse Events v4.03 (19). Other safety evaluations included death and changes in clinical laboratory measures, electrocardiogram (ECG) measures, vital signs, and ECOG performance status. Safety was assessed from screening to the end of the treatment visits.

Efficacy endpoints included best overall response [BOR: complete response (CR), partial response (PR), stable disease (SD), or progressive disease] according to the RECIST v1.1. criteria (20), clinical benefit (defined as CR, PR, or SD for ≥12 weeks) and progression-free survival (PFS). PFS was defined as time from first dose to objective disease progression or death, whichever occurred first. CT scans or MRI were performed predose to document the baseline tumor status using the most appropriate criteria for the malignancy type. Target and non-target lesions were selected on the basis of the initial scan. Clinical efficacy was assessed by the investigators according to RECIST v1.1 every two cycles (starting on day 1 of cycle 3).

Blood samples for M8891 pharmacokinetic analysis were collected on cycle 1, day 1 at 0 hour (predose within 60 minutes prior to treatment administration) and at 1, 2, 3, 4, 5, 6, 8, and 12 hours postdose; on cycle 1, day 8 at 0 hour (predose within 60 minutes prior to treatment administration); on cycle 1, day 15 at 0 hour (predose within 60 minutes prior to treatment administration) and at 1, 2, 3, 4, 6, 8, and 12 hours postdose; and on cycle 1, day 16 at 24 hours postdose (±60 minutes). From cycle 2 onward, blood samples for pharmacokinetic analysis were collected on day 1 at 0 hour (predose within 60 minutes prior to treatment administration). Validated LC/MS-MS bioanalytical methods were used to quantify concentrations of M8891 in plasma samples obtained from these blood samples. Pharmacokinetic parameters evaluated included observed maximum plasma concentration (*C*_max_), area under the concentration–time curve (AUC) using the trapezoidal rule from time zero to the last sampling time at which the concentration was at or above the lower limit of quantification (AUC_0−t_), area under the concentration–time curve from time zero to the end of the dosing period (AUC_0−tau_), and time to reach maximum observed plasma concentration (*T*_max_).

Exploratory objectives included measuring the levels of the pharmacodynamic biomarker, methionylated elongation factor 1α (Met-EF1α), before and during M8891 treatment. Tumor biopsies were collected during the screening period and on cycle 2, day 1. White blood cell (WBC) samples were collected during the screening period, predose on days 1, 2, 8, and 15 of cycle 1 and postdose on days 1 and 15 of cycle 1. In addition, WBC samples were collected predose on days 1 and 15 of cycle 2.

### Statistical Analyses

No formal significance level was defined for this study and all analyses were considered descriptive. Five analysis sets were defined: the dose-escalation set included all patients treated in the dose-escalation cohorts who did not miss >4 cumulative days of planned M8891 doses in the first cycle unless they experienced a DLT; the safety analysis set included all patients who received at least one dose of M8891; the pharmacokinetic analysis set included all patients in the safety analysis set without major protocol deviations/violations or events that would affect pharmacokinetics; the pharmacodynamics in WBCs set included all patients who received at least one dose of M8891 and provided a predose and at least one postdose WBC assessment; and the pharmacodynamics in tumor tissue set included all patients who received at least one dose of M8891 and provided tumor tissue at baseline and at the postdose tumor assessment.

To assist the SMC in making recommendations on the next dose level and the MTD, the posterior probabilities (2.5%, 25%, 50%, 75%, and 97.5% quantiles) of toxicity were estimated by a BLRM with overdose control. The model was updated with the number of evaluable patients and DLTs observed after completion of the DLT period for each cohort. The target toxicity for the suggested MTD by the BLRM was 30%.

For the efficacy analysis, objective response and clinical benefit were summarized and 95% exact Clopper–Pearson confidence intervals (CI) were estimated (21). Kaplan–Meier estimates were calculated for PFS and the CI for the median PFS was calculated according to Brookmeyer and Crowley (22).

Dose proportionality was assessed using the “power model”; the pharmacokinetic endpoints, AUC, and *C*_max_ of M8891 were compared between dose levels on day 1 and day 15 separately.

### Data Availability

For all new products or new indications approved in both the European Union and the United States after January 1, 2014, the healthcare business of Merck KGaA, Darmstadt, Germany will share patient-level and study-level data after deidentification as well as redacted study protocols and clinical study reports from clinical trials in patients. These data will be shared with qualified scientific and medical researchers, upon the researcher's request, as necessary for conducting legitimate research. Such requests must be submitted in writing to the company's data sharing portal. More information can be found at https://www.merckgroup.com/en/research/our-approach-to-research-and-development/healthcare/clinical-trials/commitment-responsible-data-sharing.html. Where the healthcare business of Merck KGaA, Darmstadt, Germany has a co-research, co-development, or co-marketing/co-promotion agreement or where the product has been out-licensed, it is recognized that the responsibility for disclosure may be dependent on the agreement between parties. Under these circumstances, the healthcare business of Merck KGaA, Darmstadt, Germany will endeavor to gain agreement to share data in response to requests.

## Results

### Patient Demographics and Disposition

Baseline and disease history characteristics of all 27 patients enrolled are presented in Table 1. All patients received at least one dose of M8891. Overall, 51.9% (14/27) of patients were male, 77.8% (21/27) were White, 7.4% (2/27) were Black or African American, and 11.1% (3/27) were Asian. The median (range) age of patients was 64.5 years (41.8–76.8). All patients had previously received at least one anticancer drug regimen. Patients were enrolled into one of six dose levels: 7, 12, 20, 35, 60, and 80 mg once daily M8891.

**TABLE 1:** Patient demographics and disposition

Characteristic	Total *n* = 27 (100%)
Sex	*n* (%)
Male	14 (51.9)
Female	13 (48.1)
Race	
White	21 (77.8)
Black or African American	2 (7.4)
Asian	3 (11.1)
Other	1 (3.7)
Age (years)	
Mean ± StD	63.5 ± 8.39
Median	64.5
Min; max	41.8; 76.8
Primary location	
Colon/rectum	8 (29.6)
Pancreas	4 (14.8)
Female reproductive tract	3 (11.1)
Liver	2 (7.4)
Lung	2 (7.4)
Stomach	2 (7.4)
Skin	2 (7.4)
Head and neck	2 (7.4)
Breast	1 (3.7)
Parotid gland	1 (3.7)
Indications	
Adenocarcinoma	21 (77.8)
Colorectal	8 (29.6)
Pancreatic	4 (14.8)
Stomach	2 (7.4)
Other^a^	7 (25.9)
Epithelioid hemangioendothelioma i	1 (3.7)
Hepatocellular carcinoma i	1 (3.7)
Merkel cell carcinoma i	1 (3.7)
Neuroendocrine i	1 (3.7)
Squamous cell carcinoma ii	2 (7.4)
Tonsil	1 (3.7)
Ear	1 (3.7)
Prior cancer treatment/s	
1	2 (7.4)
2	2 (7.4)
3	7 (25.9)
≥4	16 (59.3)
ECOG performance status at baseline	
0	12 (44.4)
1	15 (55.6)

Abbreviations: ECOG, Eastern Cooperative Oncology Group; Max, maximum; Min, minimum; StD, standard deviation.

^a^One each of breast, cervical, liver, maxillary sinus, ovarian, parotid gland, and uterine adenocarcinoma.

Thirteen of 27 patients (48.1%, *n* = 3 at 7 mg once daily, *n* = 1 at 12 mg once daily, *n* = 2 at 20 mg once daily, *n* = 3 at 35 mg once daily, *n* = 2 at 60 mg once daily, *n* = 2 at 80 mg once daily) were treated for 12–133 days until disease progression, 7 patients (25.9%, *n* = 2 at 12 mg once daily, *n* = 1 at 20 mg once daily, *n* = 2 at 35 mg once daily, *n* = 2 at 60 mg once daily) were treated for 14–140 days until withdrawal of consent due to the development of TEAEs, and another 7 patients (25.9%, *n* = 3 at 35 mg once daily, *n* = 3 at 60 mg once daily, *n* = 1 at 80 mg once daily) were treated for 6–49 days until development of TEAEs that led to treatment discontinuation.

### Safety

Overall, most patients (96.3%, 26/27) experienced a TEAE, with 21 (77.8%) reporting at least one TEAE considered related to M8891 by investigators (Table 2). Eight (29.6%) patients experienced serious TEAEs, of whom 1 patient experienced a M8891-related serious TEAE (Table 2).

**TABLE 2:** Overview of TEAEs for M8891

Safety analysis set	7 mg QD *n* = 3	12 mg QD *n* = 3	20 mg QD *n* = 3	35 mg QD *n* = 8	60 mg QD *n* = 7	80 mg QD *n* = 3	All patients *n* = 27
All TEAEs	3 (100)	2 (66.7)	3 (100)	8 (100)	7 (100)	3 (100)	26 (96.3)
M8891-related	2 (66.7)	1 (33.3)	2 (66.7)	6 (75.0)	7 (100)	3 (100)	21 (77.8)
Serious TEAEs	0 (0.0)	1 (33.3)	3 (100)	3 (37.5)	1 (14.3)	0 (0.0)	8 (29.6)
M8891-related	0 (0.0)	0 (0.0)	0 (0.0)	0 (0.0)	1 (14.3)^a^	0 (0.0)	1 (3.7)
TEAEs leading to discontinuation	0 (0.0)	0 (0.0)	0 (0.0)	3 (37.5)	3 (42.9)	1 (33.3)	7 (25.9)
**Dose-escalation set**	**7 mg QD** ***n* = 3**	**12 mg QD** ***n* = 3**	**20 mg QD** ***n* = 3**	**35 mg QD** ***n* = 5**	**60 mg QD** ***n* = 5**	**80 mg QD** ***n* = 2**	**All patients** ***n* = 20**
DLTs	0 (0.0)	0 (0.0)	0 (0.0)	0 (0.0)	1 (20.0)	1 (50.0)	2 (10.0)
Platelet count decrease	0 (0.0)	0 (0.0)	0 (0.0)	0 (0.0)	1 (20.0)	1 (50.0)	2 (10.0)

Abbreviations: TEAE, treatment-emergent adverse event; QD, once daily.

^a^Platelet count decrease that was also classified as a DLT and led to M8891 discontinuation.

TEAEs occurring in ≥15% patients are presented by dose level and overall, in Table 3. The most common TEAE (of any grade) was platelet count decrease or thrombocytopenia, which occurred in a total of 13 patients (48.2%): of those, 11 patients (40.7%) experienced platelet count decrease, with grade ≥3 platelet count decrease observed in the 60 mg once daily (*n* = 4, 14.8%) and 80 mg once daily (*n* = 2, 7.4%) dose groups; another two events (7.4%) were reported as thrombocytopenia (one of which was grade 3, in the 20 mg once daily dose group).

**TABLE 3:** TEAEs occurring in ≥15% of patients overall by dose level

	Any grade							Grade ≥3
	7 mg QD *n* = 3	12 mg QD *n* = 3	20 mg QD *n* = 3	35 mg QD *n* = 8	60 mg QD *n* = 7	80 mg QD *n* = 3	Overall *n* = 27	Overall *n* = 27
TEAE	Number (%) of patients							
Platelet count decrease	0 (0.0)	1 (33.3)	2 (66.7)^a^	2 (25.0)	6 (85.7)^a^	2 (66.7)	13 (48.2)^b^	5 (18.5)^a^
Decreased appetite	1 (33.3)	1 (33.3)	1 (33.3)	3 (35.7)	1 (14.3)	2 (66.7)	9 (33.3)	0 (0.0)
Anemia	1 (33.3)	1 (33.3)	2 (66.7)	3 (37.5)	1 (14.3)	0 (0.0)	8 (29.6)	4 (14.8)
Nausea	1 (33.3)	1 (33.3)	0 (0.0)	3 (37.5)	3 (42.9)	0 (0.0)	8 (29.6)	0 (0.0)
AST increase	0 (0.0)	1 (33.3)	1 (33.3)	4 (50.0)	2 (28.6)	0 (0.0)	8 (29.6)	1 (3.7)
Fatigue	0 (0.0)	1 (33.3)	2 (66.7)	2 (25.0)	1 (14.3)	1 (33.3)	7 (25.9)	0 (0.0)
ALT increase	0 (0.0)	1 (33.3)	0 (0.0)	2 (25.0)	3 (42.9)	0 (0.0)	6 (22.2)	2 (7.4)
Headache	0 (0.0)	0 (0.0)	1 (33.3)	2 (25.0)	1 (14.3)	2 (66.7)	6 (22.2)	0 (0.0)
Abdominal pain	1 (33.3)	1 (33.3)	1 (33.3)	1 (12.5)	0 (0.0)	1 (33.3)	5 (18.5)	0 (0.0)
Constipation	0 (0.0)	1 (33.3)	1 (33.3)	2 (25.0)	0 (0.0)	1 (33.3)	5 (18.5)	0 (0.0)
Disease progression	0 (0.0)	1 (33.3)	2 (66.7)	2 (25.0)	0 (0.0)	0 (0.0)	5 (18.5)	5 (18.5)
Weight decrease	0 (0.0)	0 (0.0)	2 (66.7)	2 (25.0)	1 (14.3)	0 (0.0)	5 (18.5)	0 (0.0)
Dizziness	1 (33.3)	0 (0.0)	0 (0.0)	0 (0.0)	2 (28.6)	2 (66.7)	5 (18.5)	0 (0.0)

Abbreviations: ALT, alanine aminotransferase; AST, aspartate aminotransferase; QD, once daily; TEAE, treatment-emergent adverse event.

^a^Includes thrombocytopenia (*n* = 1).

^b^Includes thrombocytopenia (*n* = 2).

**FIGURE 1:**
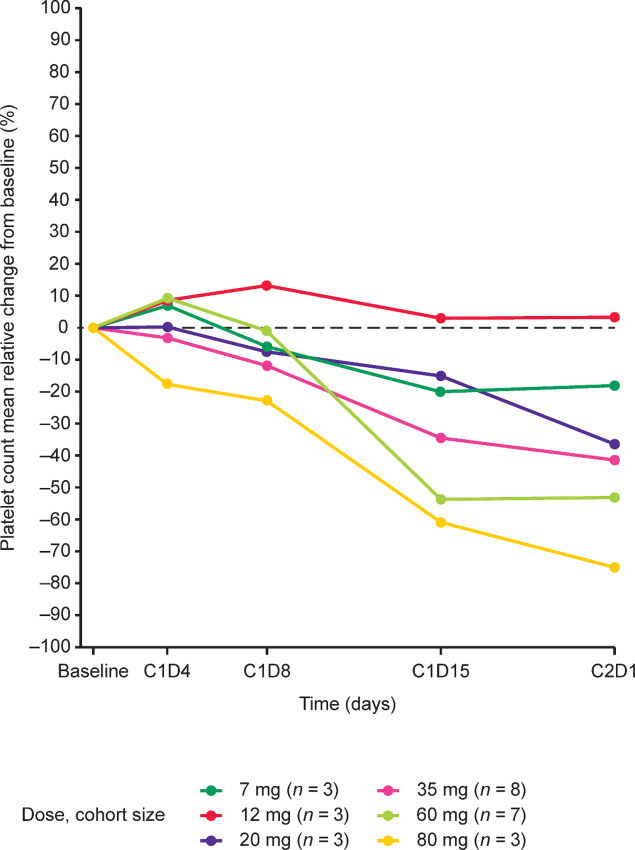
Mean relative change in platelet count from baseline per dose level in cycle 1 (%). C, cycle; D, day.

In 4 patients, platelet count decrease or thrombocytopenia led to M8891 dose interruption [1 patient each in the 20 mg (thrombocytopenia) and 80 mg once daily (platelet count decrease) dose group] or study treatment discontinuation [1 patient each in the 35 mg and 60 mg once daily dose groups (both platelet count decrease)], with 3 patients subsequently recovering during the study period. Eight patients continued treatment with M8891 without dose interruption or reduction in response to platelet count decrease. One of these patients recovered while on treatment, 3 patients recovered after the last dose of M8891, and 4 patients had not recovered by the end of the study. In 1 patient in the 60 mg once daily dose group, platelet count decrease was detected 1 day after the last dose was administered; the patient subsequently recovered. The mean relative reduction in platelet count from baseline was greater with increasing dose levels (Fig. 1).

Other common TEAEs were decreased appetite (*n* = 9, 33.3%), nausea (*n* = 8, 29.6%), AST increase (*n* = 8, 29.6%), and anemia (*n* = 8, 29.6%; Table 3). M8891 was generally well tolerated. Most TEAEs were grade 1 to grade 2 in severity, unrelated to M8891, and clinically manageable.

Thirteen of 27 patients discontinued treatment due to disease progression (48.1%, all groups), 7 (25.9%) patients withdrew consent due to TEAEs, and another 7 (25.9%) patients experienced TEAEs that led to treatment discontinuation. Both, TEAEs leading to M8891 treatment discontinuation and M8891-related grade ≥3 TEAEs generally occurred more frequently at higher doses of M8891 (>20 mg once daily) than at lower doses (≤20 mg once daily). Six patients experienced TEAEs leading to study discontinuation that were considered M8891-related: deep vein thrombosis (*n* = 1, grade 2, 35 mg once daily M8891); platelet count decrease (*n* = 2, grade 3 on 35 mg once daily M8891 and grade 3 worsening to grade 4 before resolving on 60 mg once daily M8891); confusional state (*n* = 1, grade 2, 60 mg once daily M8891); ALT increase (*n* = 1, grade 3, 60 mg once daily M8891); and superficial thrombophlebitis (*n* = 1, grade 1, 80 mg once daily M8891). A total of 5 (18.5%) patients died during the study, all due to disease progression. All deaths occurred after discontinuation of M8891.

Two DLTs of grade 4 platelet count decrease were reported in the study, one each in the 60 and 80 mg groups. Both patients were initially reported as having grade 1 platelet count decrease after 7 and 14 days of treatment, respectively, and subsequently developed grade 4 platelet count decrease. The DLTs were manageable, not complicated by bleeding events, and their severity decreased within 1 week after discontinuation of M8891.

No clinically meaningful changes were observed in vital signs or ECG values from baseline to any postdose values. Most postdose laboratory tests values were rated grade 0 or 1 in severity. Three patients had shifts in baseline ECOG performance status to values ≥2.

Taking into consideration the less than dose-proportional increase in exposure combined with safety observations at doses of 60 to 80 mg, the SMC recommended to de-escalate from 80 to 35 mg to further explore this dose as a potential RPD2. As a result, the DLT probability estimation by the BLRM did not reach sufficient precision due to the limited number of DLTs and sample size, and the MTD was not determined.

### Pharmacokinetics

The pharmacokinetic parameters for M8891 for cycle 1, days 1 and 15 are presented with summary statistics in Table 4.

**TABLE 4:** Pharmacokinetic parameters of M8891 in plasma by treatment after single (cycle 1, day 1) and multiple dosing (cycle 1, day 15)

	7 mg QD *n* = 3	12 mg QD *n* = 3	20 mg QD *n* = 3	35 mg QD *n* = 8	60 mg QD *n* = 7	80 mg QD *n* = 3
Parameter	Geometric mean (geometric CV%)					
Cycle 1, day 1						
*C*_max_ (ng/mL)	514 (16.6)	934 (12.4)	1,780 (48.9)	2,960 (17.0)	3,630 (31.2)	4,760 (15.6)
AUC_0−t_ (hours*ng/mL)	9,080 (21.3)	16,300 (9.6)	34,200 (41.0)	59,400 (22.7)	71,700 (26.3)	86,900 (22.5)
AUC_0−tau_ (hours*ng/mL)	9,100 (21.2)	16,300 (9.6)	32,400 (35.7)	59,100 (21.3)	71,400 (26.8)	84,500 (20.6)
*T*_max_ (hours)^a^	3.17 (3.00–24.0)	4.00 (2.00–11.0)	6.00 (2.23–6.73)	4.04 (2.92–8.02)	7.13 (4.12–23.9)	3.38 (1.87–8.07)
	**7 mg QD** ***n* = 3**	**12 mg QD** ***n* = 3**	**20 mg QD** ***n* = 2**	**35 mg QD** ***n* = 5**	**60 mg QD** ***n* = 4**	**80 mg QD** ***n* = 2**
**Parameter**	**Geometric mean (geometric CV%)**					
Cycle 1, day 15						
*C*_max_ (ng/mL)	1,260 (39.2)	2,210 (21.1)	NC^b^	6,840 (16.9)	6,600 (20.2)	NC^b^
AUC_0−t_ (hours*ng/mL)	25,400 (50.3)	38,600 (17.6)	NC^b^	141,000 (13.9)	134,000 (18.4)	NC^b^
AUC_0−tau_ (hours*ng/mL)	25,700 (48.4)	38,500 (18.7)	NC^b^	137,000 (15.6)	133,000 (17.4)	NC^b^
*T*_max_ (hours)^a^	4.00 (3.10–4.98)	2.00 (1.10–3.00)	NC^b^ (2.83–3.97)	4.07 (1.32–8.00)	3.11 (2.05–22.8)	NC^b^ (1.98–4.18)

Abbreviations: AUC_0−t_, area under the concentration–time curve from time zero to the last sampling time at which the concentration was at or above the lower limit of quantification; AUC_0−tau_, area under the concentration–time curve from time zero to the end of the dosing period; *C*_max_, observed maximum plasma concentration; CV%, coefficient of variation; *T*_max_, time to reach maximum observed plasma concentration; NC, not calculable.

^a^Median (min, max).

^b^Not calculated because <3 values were available.

Except for a few subjects (*n* = 3), the time to reach maximum plasma concentrations ranged from approximately 2 to 8 hours postdose at day 1 (Table 4) and 1 to 6 hours postdose at day 15 (Table 4).

**FIGURE 2:**
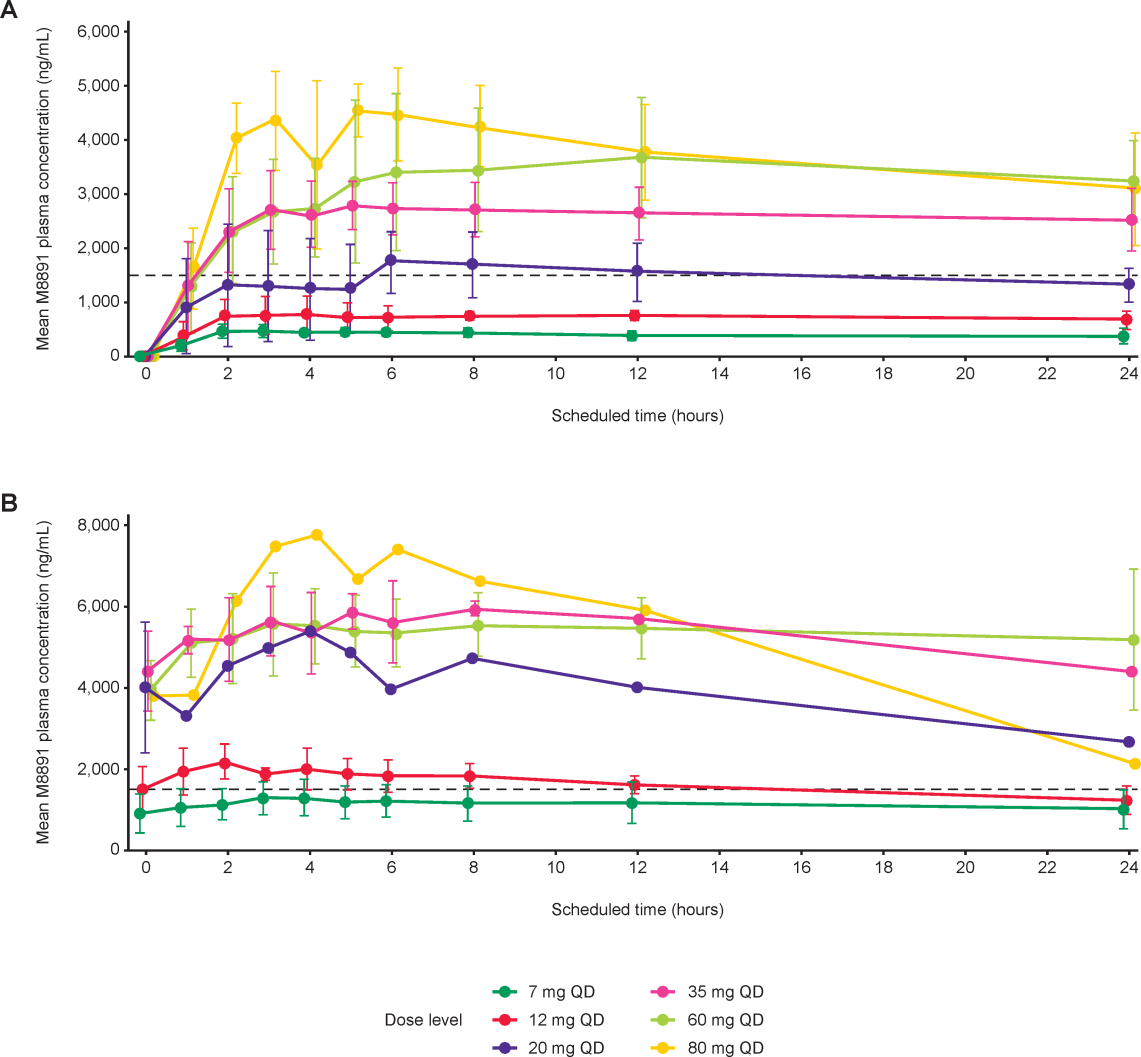
Plasma concentration–time profiles of M8891 (mean ± SD) at cycle 1, day 1 (**A**) and cycle 1, day 15 (**B**). The dashed line shows the target M8891 C_trough_ level of 1,500 ng/mL expected to be correlated with efficacy. QD, once daily.

Peak and total exposures (*C*_max_, AUC_0−t_, AUC_0−tau_) of M8891 appeared to increase approximately dose proportionally up to 35 mg; at doses greater than 35 mg, M8891 exposures appeared to increase in a less than dose-proportional manner (Fig. 2).

From day 1 to day 15 of cycle 1, M8891 showed slight accumulation across all doses. The accumulation ratio for AUC ranged from 1.99 to 2.90, in line with a terminal half-life of approximately 30 hours and once daily dosing, and the accumulation ratio for *C*_max_ ranged from 1.96 to 2.58.

Pharmacokinetic parameters after repeated dosing generally exhibited low to moderate interpatient variability, ranging from 7.7% to 51.7%. Starting with 20 mg, steady-state C_trough_ (C_ss,trough_) levels were above the target level of 1,500 ng/mL (Fig. 2) defined by modeling of preclinical pharmacokinetics/pharmacodynamics and tumor growth inhibition data (El Bawab S, personal communication).

### Pharmacodynamics

**FIGURE 3:**
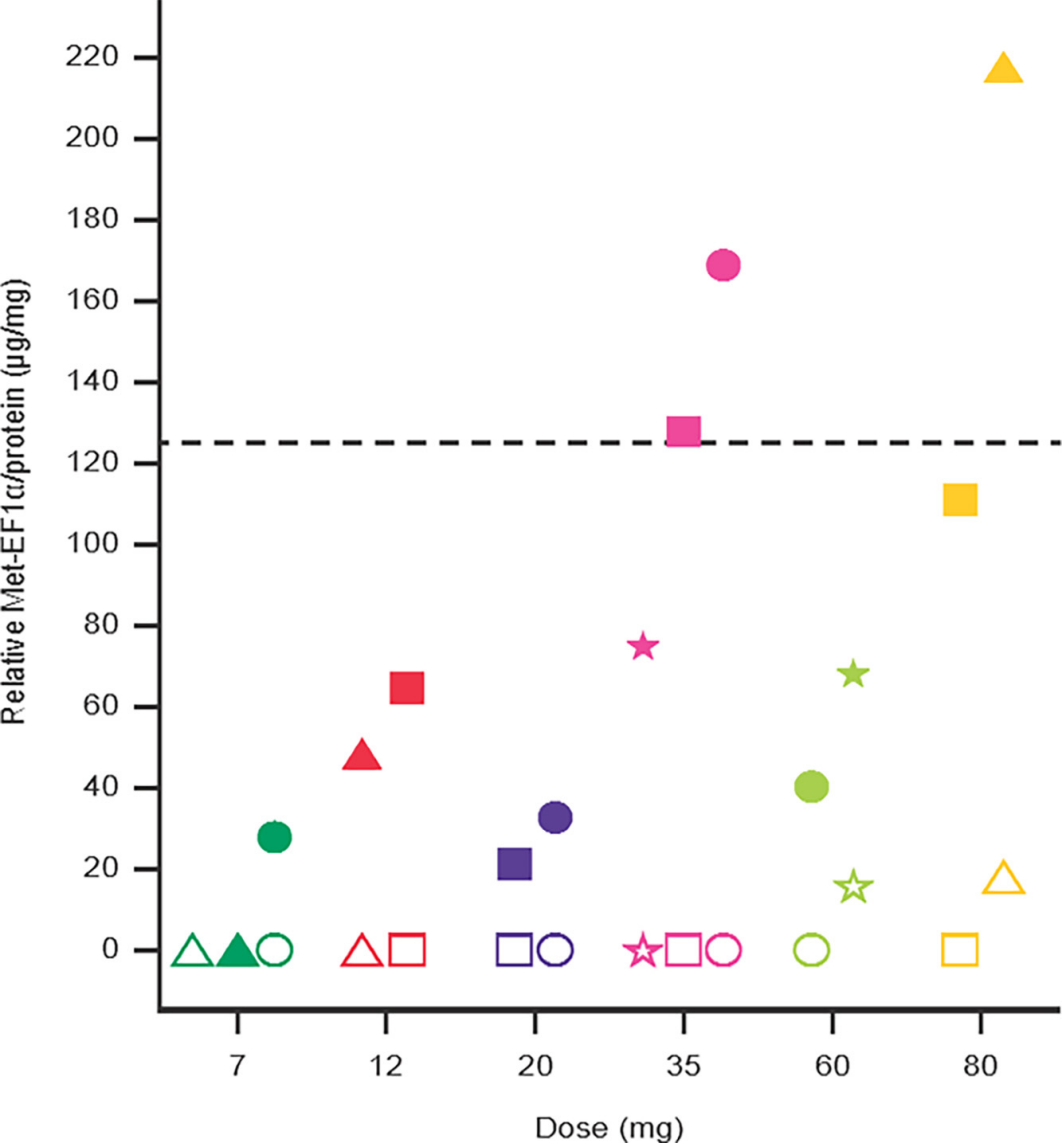
Met-EF1α/protein levels in tumor on cycle 2, day 1 (individual patient dot plot). Open symbols show the pretreatment Met-EF1α levels, and the corresponding closed symbols show cycle 2, day 1 Met-EF1α levels for individual patients for the doses of 7, 12, 20, 35, 60, and 80 mg. The dashed line shows the target Met-EF1α level of 125 μg/mg protein expected to be correlated with efficacy. Met-EF1α, methionylated elongation factor 1α.

Met-EF1α accumulation in tumors was observed at 7 mg once daily and tended to increase up to 35 mg once daily in a dose-dependent manner in line with the observed increase of exposure (Fig. 3). A small Met-EF1α signal was observed in pretreatment tumor tissue samples in 2 patients at doses of 60 and 80 mg once daily. At ≥35 mg once daily, Met-EF1α levels reached the target levels of 125 μg/mg protein required for efficacy defined by preclinical modeling (El Bawab S, personal communication).

At 12 mg once daily and higher, a low and variable pharmacodynamic response was detected in WBCs from cycle 1, day 15 onward; no obvious dose correlation was observed.

On the basis of the observed safety, pharmacokinetic, and pharmacodynamic data, the SMC suggested a dose of 35 mg once daily as the RP2D for M8891 monotherapy.

### Efficacy

**FIGURE 4:**
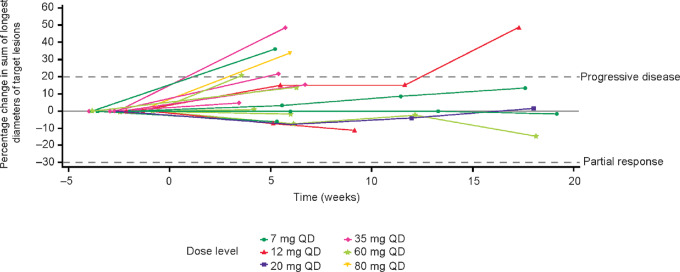
Percentage change from baseline of the sum of longest target lesion diameters. The dashed lines show thresholds for progressive disease and PR. Dose levels are represented by different colors and symbols; the different curves at each dose level represent different patients.

Although no objective responses were observed in this heavily pretreated study population, 7 (25.9%) patients had a BOR of SD (*n* = 2 at 7 mg once daily dose, *n* = 2 at 12 mg once daily, *n* = 1 at 20 mg once daily, *n* = 2 at 60 mg once daily) for 42–123 days. Three patients (11.1%; 95% CI, 5.0–53.8), 2 treated with 7 mg once daily M8891 and 1 with 60 mg once daily M8891, had SD for ≥12 weeks (Fig. 4).

## Discussion

In this phase I study, orally administered M8891 was clinically manageable in patients with advanced solid tumors. Among the 27 patients enrolled into the study, only two DLTs were observed, and, after enrolling a confirmatory cohort, the RP2D was determined as 35 mg once daily based on the absence of DLTs at doses ≤35 mg and a dose-proportional increase in M8891 exposure and Met-EF1α pharmacodynamic biomarker levels in tumors up to 35 mg once daily.

TEAEs were generally grade 1–2 in severity and most were unrelated to M8891. Platelet count decrease was the most common TEAE, but these events were generally manageable clinically, and no associated bleeding events were observed. These results compare favorably with the safety profile of irreversible MetAP2 inhibitors, such as TNP-470, which showed considerable neurotoxicity in phase I studies (23, 24), and continue to support the hypothesis that the toxicity of irreversible MetAP2 inhibitors is not related to inhibition of MetAP2 itself (12) or sufficiently compensated by redundant MetAP1 activity [see Goya Grocin 2021 (11)].

Exposure to M8891 increased by dose level and showed low-to-moderate interpatient variability following multiple doses based on pharmacokinetic parameters. The multiple-dose pharmacokinetics of M8891 compared favorably with high pharmacokinetic variability found in phase I studies with TNP-470 (25). Moreover, at doses ≥20 mg once daily, the observed C_ss,trough_ levels of M8891 were well above 1,500 ng/mL (Fig. 2), the target level anticipated to be correlated with *in vivo* efficacy (tumor growth inhibition) as defined by modeling and simulation of preclinical pharmacokinetic/pharmacodynamic and efficacy data (El Bawab S, personal communication). In short, a pharmacokinetic/pharmacodynamic model was established to describe the relationship between Met-EF1α modulation in human tumor cell xenografts in mice and M8891 mouse plasma exposure, and simulations were performed to identify the Met-EF1α levels at efficacious doses. The human target pharmacokinetic level could then be obtained by simulating the human dose and exposure leading to an accumulation of Met-EF1α similar to the level identified as linked to antitumor activity, using predicted human pharmacokinetic parameters and assuming a similar pharmacokinetic/pharmacodynamic relationship in human and in human tumor xenografts in mice.

M8891 treatment resulted in the accumulation of Met-EF1α in tumors in a dose-dependent manner, demonstrating inhibition of MetAP2 by M8891, consistent with preclinical studies (17). In addition, at doses ≥35 mg once daily, accumulation of Met-EF1α reached the target level of 125 μg/mg protein (Fig. 3) assumed to be correlated with *in vivo* efficacy as determined by preclinical modeling of pharmacokinetic/pharmacodynamic and efficacy data (El Bawab S, personal communication). It should be noted that in contrast to preclinical pharmacodynamic data in WBCs (Friese-Hamim M, personal communication), the corresponding data for human WBCs showed only a marginal pharmacodynamic response, and thus cannot be used as a surrogate for the pharmacodynamic response in tumors.

Although M8891 had shown significant tumor growth inhibition in preclinical models (17), the clinical efficacy described in this study was modest, with no objective responses observed (Fig. 4). However, the impact of the seven observed responses of SD should not be underestimated, given that a heavily pretreated, biomarker unselected patient population was treated with M8891 monotherapy. Furthermore, the low patient numbers treated at each dose level and the variability of tumor types precluded meaningful comparisons between response and dose level. Other reversible and irreversible MetAP2 inhibitors have struggled to demonstrate objective responses in phase I studies (26, 27). LAF389, a reversible MetAP2 inhibitor, failed to show any objective responses in patients with advanced solid tumors (26) and TNP-470 also failed to show any significant objective responses in a phase II study of patients with metastatic renal carcinoma (27). However, the tolerability of M8891 and promising preclinical efficacy in combination with VEGFR-targeted TKIs indicate that further investigations with M8891, especially in combination regimens, are warranted (17).

As this was a phase I dose-escalation study, limitations include the open-label design and small sample size. The heterogeneity of tumor types enrolled in the study made it challenging to assign the efficacy or pharmacodynamic data to specific patient populations that may benefit more than others.

In conclusion, M8891 is the first orally available, reversible MetAP2 inhibitor that has entered clinical development. This FIH study allowed determination of an RP2D based on relevant pharmacokinetics, pharmacodynamics, and safety observations; M8891 demonstrated a manageable safety profile with TEAEs in line with the patient population, drug effect, dose-proportional exposure up to 35 mg, Met-EF1α accumulation, and low-to-moderate interpatient variability of plasma concentration and corresponding parameters of pharmacokinetics. In addition, the observed exposure and target engagement anticipated to be required for *in vivo* efficacy were achieved at 35 mg once daily. Taken together, the RP2D of M8891 was determined as 35 mg once daily. Further investigations with M8891, especially in combination with other anticancer agents (Friese-Hamim M, personal communication), are warranted. Further development of M8891 will be managed by the Oncoteq AG subsidiary of Cureteq AG (28), which licensed M8891 from Merck KGaA following determination of the RP2D in this phase Ia trial and the strategic portfolio decision by Merck KGaA.
